# Deformation-induced grain boundary segregation in a powder-metallurgy ultrafine-grained MoNbTaTiV refractory high-entropy alloy

**DOI:** 10.1016/j.heliyon.2024.e37392

**Published:** 2024-09-03

**Authors:** Qing Liu, Xiaoguang Li, Guofeng Wang, Jun Zhu, Rui Zhan, Yongkang Liu, Wei Guan, Hang Liang, Lei Cui, Yongchang Liu

**Affiliations:** a School of Materials Science and Engineering, Tianjin University, Tianjin, 300072, China; b AECC Shenyang Liming Aero-Engine Co., Ltd., Shenyang, 110043, China; c National Key Laboratory for Precision Hot Processing of Metals, Harbin Institute of Technology, Harbin, 150006, China; d Beijing Power Machinery Research Institute, Beijing, 100071, China; e Tianjin Key Laboratory of Advanced Joining Technology, Tianjin, 300072, China

**Keywords:** Refractory high-entropy alloy, Mechanical alloying, Segregation, Hot deformation, Microstructure

## Abstract

A powder-metallurgy MoNbTaTiV refractory high-entropy alloy synthesized by mechanical alloying (MA) and spark plasma sintering was subjected to hot deformations at different temperatures and strain rates. The microstructural morphologies were characterized, and component element segregation was elucidated. With grain refinement and lattice strain increase, the large inhomogeneous milled powder became refined and homogeneous after the MA. Component element segregation was observed at relatively low deformation temperatures and high strain rates. As the deformation temperature increased and the strain rate decreased, the segregation gradually disappeared, which was attributed to dislocation movement.

## Highlights

• A powder-metallurgy MoNbTaTiV RHEA was synthesized by mechanical alloying and spark plasma sintering.
• The deformation-induced component element segregation in the refractory high-entropy alloy was elucidated.
• Effects of deformation temperature and strain rate on the deformation-induced grain boundary segregation were obtained.

## 1 Introduction

Refractory high-entropy alloys (RHEAs) are novel high-temperature structural materials based on the design concept of high-entropy alloys (HEA) [[1], [2], [3]]. The multi-principal element characteristic of RHEAs leads to unique microstructures and remarkable properties, such as extremely high melting points, excellent high-temperature strengths, outstanding thermal stabilities, and exceptional creep, corrosion, and oxidation resistances [[4], [5], [6], [7]]. Owing to these advantages, RHEAs not only exhibit a significant application potential in aerospace and nuclear energy industries as high-temperature load-bearing or thermal protection structures, but also are regarded as an important development direction of high-temperature structural materials.

Considering the crucial influence of microstructures on the properties, microstructure control is a meaningful approach to optimize and improve the macroscopic properties of alloys [6,7]. The element grain boundary segregation structure proposed in recent years provides extraordinary advantages to enhance the properties of alloys [8,9]. The interaction of segregated alloying elements and dislocation can enhance the hardness and strength of alloys. On the other hand, low-melting-point elements segregated at grain boundaries can promote grain boundary sliding deformation during hot forming, achieving superplastic deformation behaviors [10]. Hence, the introduction of elemental grain boundary segregation structures into ultrafine-grained RHEAs could be an excellent strategy to improve performance characteristics and enrich the microstructure control range.

Severe plastic deformation (SPD) is an effective approach to induce component element grain boundary segregation [11]. Both dislocations and defects generated during the deformation supply a driving force for the segregation behavior of solute atoms. The SPD techniques, such as surface mechanical grinding treatment and high-pressure torsion, have been successfully applied for Al and Mg alloys to obtain the grain boundary segregation structure and corresponding expected properties [12,13]. Notably, these methods are based on a considerable plasticity and acceptable deformation resistance at room temperature. However, for the ultrafine-grained RHEAs, the exceedingly high strength and limited ductility have brought considerable challenges to the SPD process. An increase in deformation temperature and reduction in strain rate are useful to improve the deformation performance. A higher temperature and lower strain rate are also conducive to a more adequate diffusion of solute atoms.

Hence, based on the hot deformation technique and admissible plasticity of MoNbTaTiV RHEAs, the interaction between dislocation movement and multi-principal elemental atoms was used to obtain the grain boundary segregation structure in ultrafine-grained RHEAs, and the effect of segregation on the properties was explored. The findings could provide a new approach for the on-demand optimization of the microstructures of RHEAs and be of significance for their engineering applications.

## 2 Experiment and methods

High-purity elemental powders of Mo (99.9 %, ∼2 μm), Nb (99.9 %, ∼48 μm), Ta (99.9 %, ∼48 μm), Ti (99.5 %, ∼74 μm), and V (99.9 %, ∼48 μm) were used as original materials. A nanocrystalline MoNbTaTiV RHEA powder with homogeneous component element distributions was synthesized through mechanical alloying (MA) at the solid state, and subsequently spark-plasma-sintered at 1873 K for 10 min under a vacuum of 1.3 × 10^−2^ Pa. The hot compression deformations were performed at 1373, 1473, and 1573 K at strain rates of 0.0005, 0.005, and 0.05 s^−1^, which represented the lower, medium and higher hot processing procedures, respectively. Vickers hardness tests were conducted at a load of 9.8 N with a loading time of 15 s. The microstructures of MoNbTaTiV RHEAs were characterized by backscattered electron (BSE) and transmission electron microscopy (TEM) techniques. The duplicate samples for TEM observation were cut by the wire-EDM method, having a diameter of 3 mm. Subsequently, these were mechanically ground down to 50 μm and thinned through the Gatan PIPS II.

## 3 Results and discussion

**Fig. 1:**
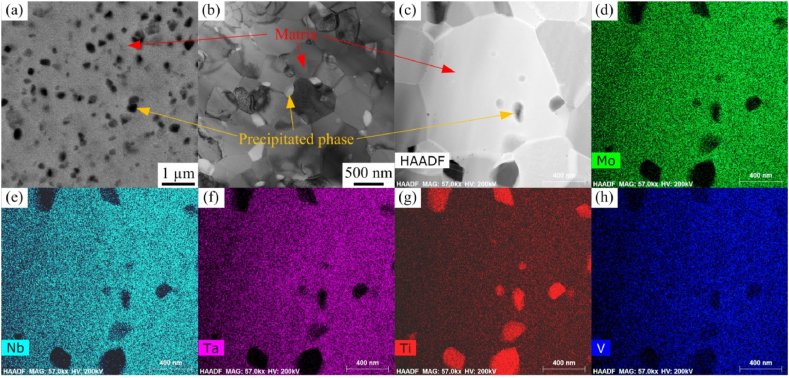
Characterization of the sintered MoNbTaTiV RHEA: (a) BSE image, (b) TEM bright-field image, (c) high-angle annular dark-field (HAADF) image, and (d)–(h) Mo, Nb, Ta, Ti, and V component distributions.

The microstructures of the sintered MoNbTaTiV RHEA are presented in Fig. 1. The ultrafine-grained MoNbTaTiV RHEAs consisted of the matrix with an average grain size of 0.58 μm and smaller precipitated phase with an average size of 0.18 μm. Component elements were uniformly distributed in the matrix. The induced precipitated phase during the MA process was mainly a compound of Ti, dispensed inside matrix grains and on matrix grain boundaries.

**Fig. 2:**
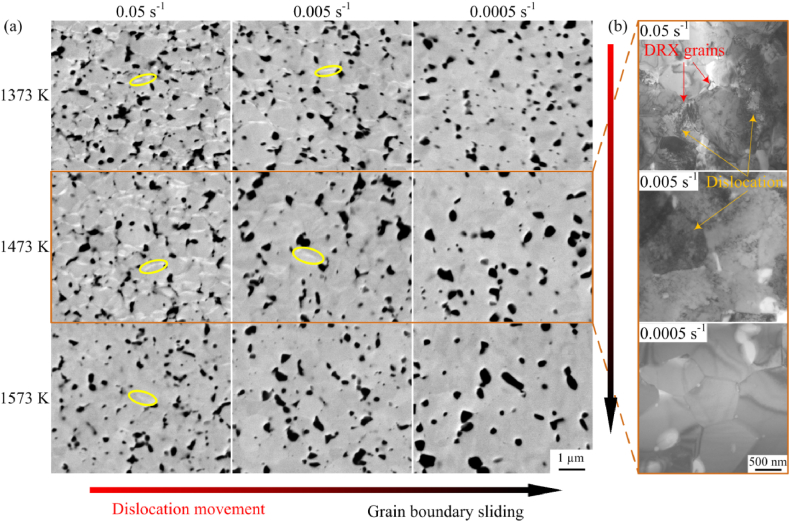
Microstructures of deformed MoNbTaTiV RHEAs: (a) BSE images after deformations and (b) TEM bright-field images after deformations at 1473 K at different strain rates.

BSE images of the ultrafine-grained MoNbTaTiV RHEA after the deformations are shown in Fig. 2(a). It can be seen that, compared with the microstructures in the arc melting and additively manufactured RHEAs, there existed the finer grains in the powder-metallurgy alloys [14,15]. The obvious component element segregation phenomenon of white banding (marked by yellow circles) is observed for deformations at relatively low temperatures and high strain rates. As the deformation temperature increased and the strain rate decreased, the segregation gradually disappeared. In the BSE image, the intensity of an element is approximately proportional to the atomic number. These white bands indicate heavier atoms among the constituent elements of the MoNbTaTiV RHEA, as verified by TEM. The hot deformation behaviors of the ultrafine-grained MoNbTaTiV RHEA were extremely sensitive to the deformation temperature and strain rate. The dominant deformation mechanism at low temperatures and high strain rates was dislocation movement, transformed to grain boundary sliding with the increase in temperature and reduction in strain rate. A large number of dislocations entanglements and nanocrystalline dynamic recrystallization (DRX) grains are observed at 1473 K and 0.05 s^−1^ in the TEM bright-field images in Fig. 2(b). When the strain rate gradually decreased to 0.0005 s^−1^, the dislocation density inside the grain gradually diminished until it disappeared, and the grain boundaries flattened. Thus, the component element segregations were closely related to the dislocation movements.

**Fig. 3:**
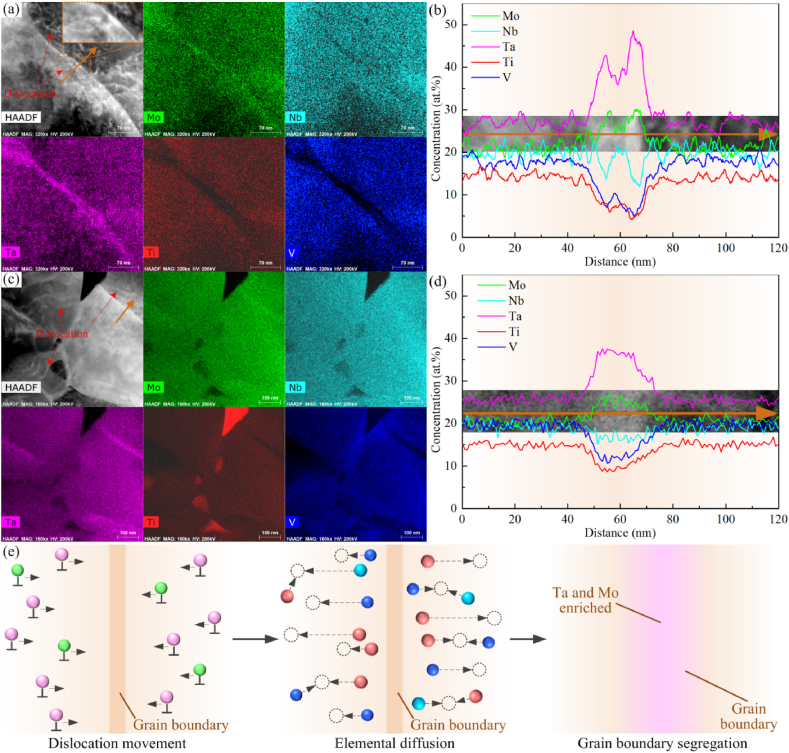
(a) Component elemental mappings under deformation conditions of 1373 K and 0.05 s^−1^, (b) elemental line distributions for the yellow line in (a), (c) component elemental mappings under deformation conditions of 1473 K and 0.05 s^−1^, (d) elemental line distributions for the yellow line in (c), and (e) schematic of the elemental segregation.

The component element distributions in the ultrafine-grained MoNbTaTiV RHEA are presented in Fig. 3(a–d). More Ta elements and some Mo elements were segregated at grain boundaries of matrix grains. On the contrary, the elements of Ti and V were reduced evidently at the same locations; the Nb element is also, to some extent, lacking. These component elements are homogeneously distributed inside matrix grains, which indicates that the fluctuation of element concentration occurred only near grain boundaries. Fig. 3(b) and (d) show results of line scans marked by yellow lines in Fig. 3(a) and (c). Obvious dislocations are observed near grain boundaries, which are the dominant driving force for the movement of Ta and Mo atoms. Under the drag of the movement of intragranular dislocations, the Ta and Mo atoms in two adjacent grains gather to grain boundaries, and form double high-concentration peaks on the sides of the grain boundaries before arriving at the grain boundaries (Fig. 3(b)). With the dislocations further moving to the grain boundary, the high concentration of Ta and Mo atoms of double peaks on both sides of the grain boundary merges into a single peak at the grain boundary (Fig. 3(d)).

Different from the plastic deformation at room temperature, atoms were in a thermally activated state at a higher temperature and had a reduced potential barrier of motion. When the macroscopic deformation resulted in generation and movement of dislocations, the atoms of some elements easily followed the movement under the stress field generated by the slip and climb of the dislocations and accumulated as the dislocations annihilated at grain boundaries. The characteristics of multi-principal elements in RHEAs made the interaction between atoms more complex and supplied various aisles. On the other hand, the internal energy stored in the alloys by the plastic deformation increased the instability of component atoms. It indirectly promoted movement and diffusion of atoms. With the increasing temperature and decreasing strain rate, the intragranular dislocation generation was limited owing to the gradually enhanced grain boundary sliding mechanism. The dislocation movement also slowed. Meanwhile, the component element segregation disappeared. The accumulation of Ta and Mo atoms near grain boundaries not only occupied most positions of the lattices but also resulted in many intragranular atom vacancies. The elements of Ti and V with relatively low melting points and weak bonding force, as well as higher diffusion rates, are forced to diffuse into the crystal against the concentration gradient. Moreover, the atoms near vacancies in grains interfused. Under the combined action of these factors, a mass of Ta atoms and some Mo atoms were segregated at grain boundaries with a larger width, but the concentration of Ti and V decreased, and there was a slight reduction in Nb. The distributions of all component elements in the grains were still uniform. A schematic of these segregation processes is presented (Fig. 3(e)).

**Fig. 4:**
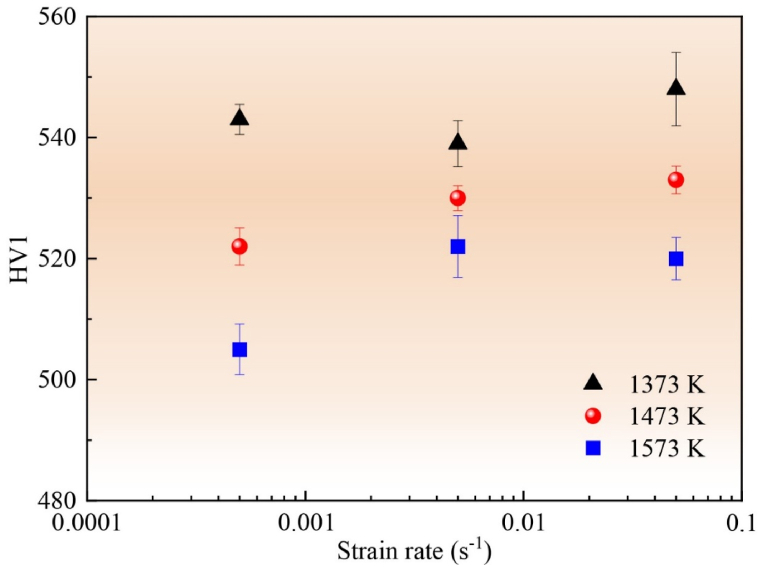
Microhardness comparison of ultrafine-grained MoNbTaTiV RHEAs under different deformation conditions.

The microhardnesses of ultrafine-grained MoNbTaTiV RHEAs after different hot deformations are compared (Fig. 4). In general, under the various strain rates, the microhardness decreased with the hot deformation temperature increase. The difference in hardness at 0.005 s^−1^ was smaller than those at 0.0005 and 0.05 s^−1^. For certain temperature, as the strain rate increased during the hot deformation, there existed a nonmonotonic variation in microhardness.

## 4 Conclusions

Ultrafine-grained MoNbTaTiV RHEAs were obtained via MA, spark plasma sintering, and hot deformation at various high temperatures and strain rates. With grain refinement and lattice strain increase, the large and inhomogeneous milled powder became refined and homogeneous after the MA. The component elements were segregated under relatively low temperatures and high strain rates. The fluctuation of element concentration occurred only near grain boundaries. The segregation gradually disappeared with the increase in deformation temperature and decrease in strain rate. The interaction between multi-principal elements and dislocation movement was responsible for component element segregation and determined the microhardness variations.

## Ethics declarations

Review and/or approval by an ethics committee was not needed for this study because this paper is a review article. Ethics committees and Informed consent are not involved.

## Data availability statement

Our study has not been deposited into a publication available repository. Data will be made available on request.

## CRediT authorship contribution statement

**Qing Liu:** Writing – original draft, Investigation, Funding acquisition, Data curation. **Xiaoguang Li:** Writing – original draft, Investigation, Formal analysis. **Guofeng Wang:** Writing – review & editing, Supervision, Funding acquisition. **Jun Zhu:** Investigation, Data curation. **Rui Zhan:** Formal analysis, Data curation. **Yongkang Liu:** Formal analysis, Validation. **Wei Guan:** Writing – review & editing, Formal analysis. **Hang Liang:** Writing – review & editing, Methodology, Funding acquisition. **Lei Cui:** Writing – review & editing, Supervision, Conceptualization. **Yongchang Liu:** Supervision, Methodology.

## Declaration of competing interest

The authors declare that they have no known competing financial interests or personal relationships that could have appeared to influence the work reported in this paper.
